# Data on characterization and performance of aspartic acid functionalized graphene oxide-polysulfone mixed matrix membranes

**DOI:** 10.1016/j.dib.2020.106197

**Published:** 2020-08-19

**Authors:** Omnya Abdalla, Md A. Wahab, Ahmed Abdala

**Affiliations:** a Chemical Engineering Program, Texas A&M University at Qatar, Doha, Qatar

**Keywords:** Mixed matrix membrane, Functionalized graphene oxide, Aspartic acid, Polysulfone, Young's Modulus, Pure water flux

## Abstract

Structure and microstructure characterization are very important for determining the membrane hydrophilicity, morphology, porosity, and mechanical properties that affect the membrane separation performance and durability. This article analyzes the morphological differences between graphene oxide (GO) and functionalized graphene oxide (f-GO) based on data from SEM imaging. nalysis of the morphology of mixed matrix membrane (MMMs) through SEM, mechanical properties through DMA and hydrophilicity through contact angle data. This dataset will support the original research article “Mixed matrix membranes containing aspartic acid functionalized graphene oxide for enhanced oil-water emulsion separation” (Abdalla, Wahab and Abdala, 2020; doi.org/10.1016/j.jece.2020.104269). The data reported here also include raw data for stress vs strain measurements, mass recorded during permeation test and pure water fluxes at different pressures for all prepared MMMs.

**Specifications Table**

**Table:** 

Subject	Materials science, nanotechnology, environmental science
Specific subject area	Membranes characterization and testing
Type of data	Tables and Figures
How data were acquired	The microstructural characterization was carried out by scanning electron microscope (SEM, FEI Quanta 400 SEM, Thermo Fisher Sci.). The transmission electron microscopy (TEM) (FEI TECNAI G2 TF20, Thermo Fisher Sci.). Hydrophilicity was tested by contact angle measurements (CA) using (Kruse drop shape analyser DSA25). Mechanical properties were also tested using uniaxial tensile testing using Dynamic mechanical analysis (DMA Q800, TA Instruments, New Castle, DE, USA). Other tests including permeation fluxes were carried out in a dead-end cell connected to a nitrogen gas cylinder that supplies the required pressure to pass water through membrane into a balance that records the mass of water as time passes.
Data format	Fitted curves and analysed data
Parameters for data collection	For the SEM images, the conditions were a chamber pressure of 1.3 mbar and very high vacuum conditions and a voltage of 30 kV. The contact angle measurements were carried out using a 2 µl sessile DI water drop placed on top of the membrane surface at room temperature, the size of membrane sheets was 4 × 4 cm (W × L). The membranes sheets used for the DMA were cut to dimensions of 5 × 30 mm (W × L) and tested at 25°C and constant speed of 500 µm/min until breakage. For the permeability tests, the test was carried out at different pressures 1, 2 and 3 bars.
Description of data collection	The SEM images were captured after placing the samples on SEM stub and coating the sample with gold. Multiple magnifications were captured for each sample. The contact angle is recorded after 5 s from placing the water droplet on the surface of the membrane. Readings were recorded for each image using the software. For the DMA, membrane was cut into the required specimen dimension (5 × 0.5 cm) using a rectangular die-cutter. The specimen was stretched at constant strain of 0.5 mm/min and the force is measured. The stress Vs. strain data are recorded and plotted for each sample. Young's modulus was calculated from the initial slope of the stress-strain curve. The permeability test was carried out using a dead-end cell. The cell used was connected to a nitrogen (N_2_) gas cylinder from the top to provide the necessary pressure to press the DI water through membrane placed at the bottom of cell. From the other side, DI water leaves the cell into a beaker placed on the top of a scale that records the mass of water as time passes and saves it into a computer.
Data source location	Materials Lab, Chemical Engineering Program, Texas A&M University at Qatar Central Material Facility- Texas A&M University at Qatar. Core Labs- Qatar Environment and Energy Research Institute. Central Laboratory Unit- Qatar University.
Data accessibility	All data are available with this article
Related research article	Mixed matrix membranes containing aspartic acid functionalized graphene oxide for enhanced oil-water emulsion separation. https://doi.org/10.1016/j.jece.2020.104269

**Value of the Data**

• The data confirms the structure-property correlation of the PS-fGO MMMs by raw SEM, DMA and contact angle results, such raw data are rarely found in literature.
• These data are valuable because the incorporation of fGO into the PS membranes has significantly enhanced the membrane permeabilitys by more than 95%.
• The raw data provided here could benefit numerous researchers who study nanomaterial and polymers. As the effect of incorporating nanomaterials into polymer matrix is presented in terms of performance and characteristics. For the example, the mechanical properties data here do not provide the results of the modulus, strength, and the ductility for different samples, but provide the raw stress-strain data. These data are useful to understand the stress-strain profile. It is also useful for those who is interested in modeling the elastic properties of composite materials.
• Similarly, the presented data for permeated water versus time can be used in theoretical simulations or CFD studies for developments of models to describe the time dependence of the permeability through membranes.
• Data on the dependence of the water flux on pressure is important to analyze the impact of fGO on the membrane compression.
• PS/fGO MMMs provided significant enhancement in separation performance and mechanical properties at very low fGO concentration that does not affect the rheology of PS-fGO formulation and therefore requires no alteration to the commercial phase inversion process. Thus, great potential for optimization of ultrafiltration membranes in industrial applications.

## 1 Data Description

**Fig. 1:**
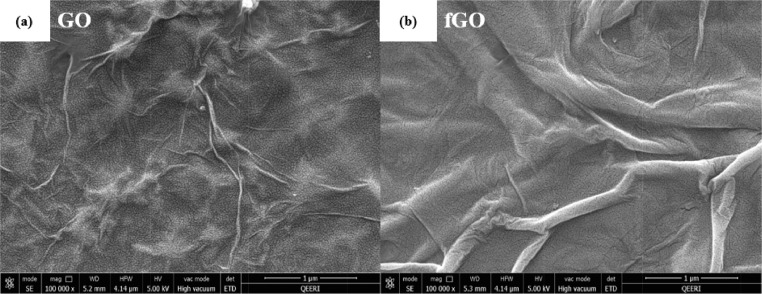
SEM images: (a) GO and (b) GO functionalized GO with aspartic acid (fGO).

**Fig. 2:**
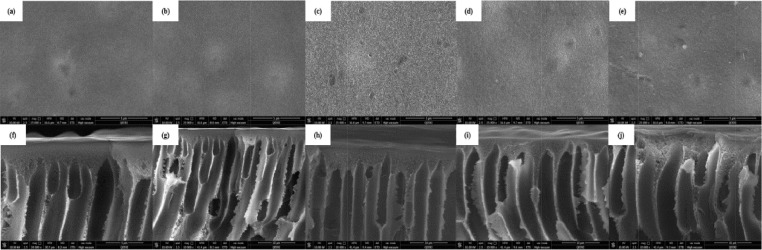
Surface and cross section SEM images of PS-fGO membranes containing (a, f) 0.05%, (b, g) 0.1%, (c, h) 0.2 %, (d, h) 0.4%, and (c, i) 0.8 % fGO, by weight.

**Fig. 3:**
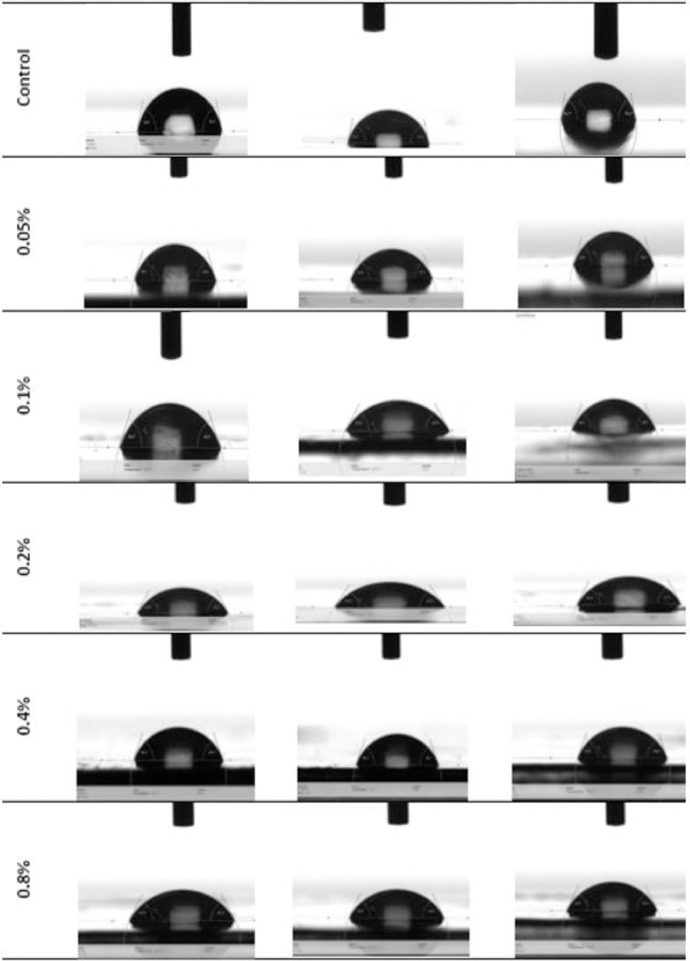
Optical images of water droplet on the surface of different PS-fGO MMMs during contact angle measurements.

**Fig. 4:**
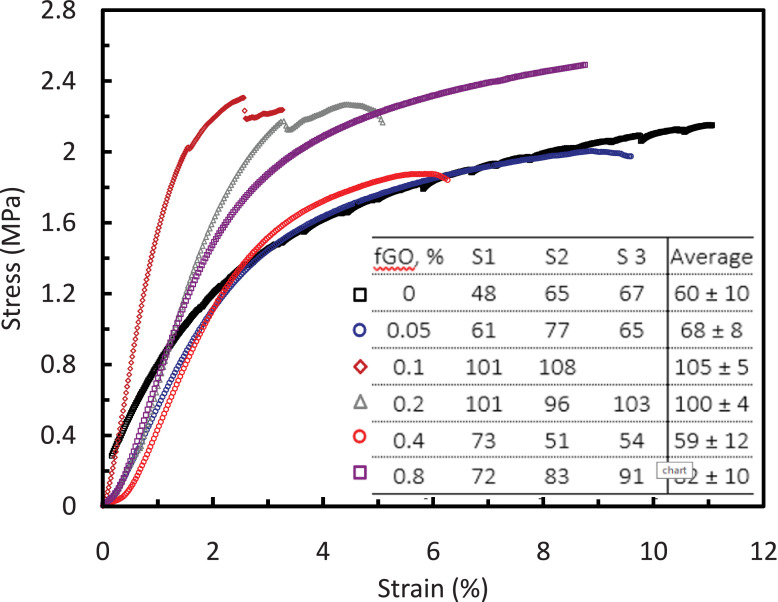
Stress vs. strain for prepared fGO membranes. Inset shows the Young's modulus for different specimens.

**Fig. 5:**
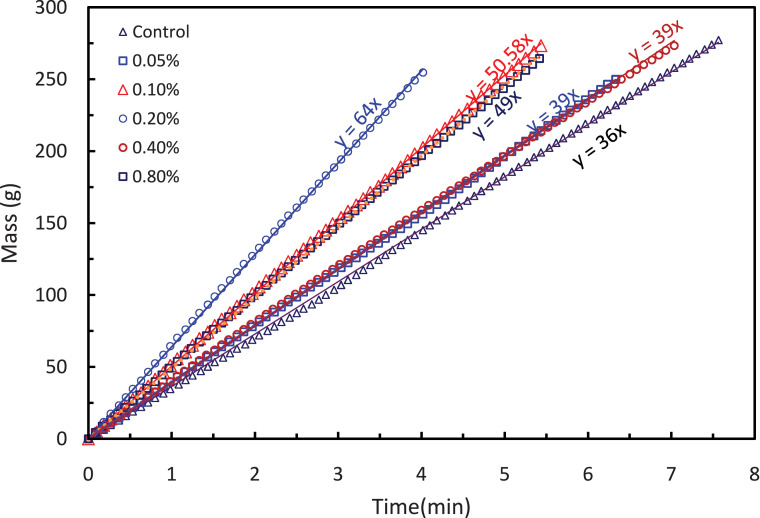
Collected mass as time passes for all PS-fGO memebranes measured at cross membrane pressure of 2 bar (run1).

**Fig. 6:**
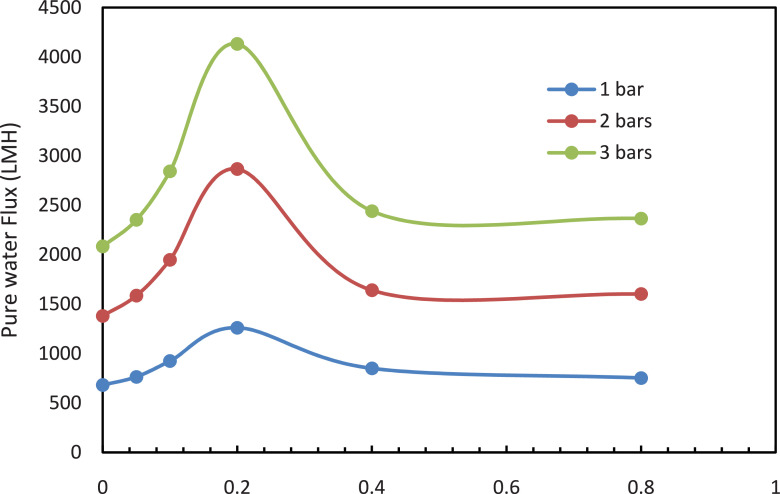
Effect of fGO concentration on permeability PS-fGO membranes measured at different pressure.

The data described in this manuscript show characterization and testing results of fGO/PS membranes [1]. Fig. 1 displays the morphology of (a) pure GO and (b) fGO, functionalized with aspartic acid. Fig. 2 displays the surface and cross-sectional SEM images of PS-fGO membranes with various fGO concentrations. Fig. 3 indicates the different contact angle measurements recorded for all the fGO membranes. Fig. 4 represents the stress vs. strain curves for one of the three runs for each the prepared membranes. Inset of Fig. 4 provides the modulus of different specimens for all membranes. Fig. 5 illustrates the mass of permeatedwater as function of time Fig. 6 shows the flux data collected at different pressure for all prepared PS-fGO membranes.

## 2 Experimental Design, Materials and Methods

### 2.1 Materials

Aspartic acid (AA), hydrochloric acid (HCl), dimethylacetamide (DMAc), Polysulfone (PS: Mn ∼ 22,000), polyvinylpyrrolidone (PVP: Mw ∼ 55,000). Graphene oxide (GO; SE2430) from Sixth Element (China).

### 2.2 Experimental Design and Methods

#### 2.2.1 Functionalization of GO (fGO)

The functionalization process of GO with Aspartic acid is completely described in our published work [1]. The process was carried out at room temperature by stirring 3 g of Aspartic acid with 3 mg/ml GO solution in 500 ml glass beaker for 5 days. After that, resulting solution was treated with HCl to convert the carboxylate groups into carboxylic acid groups. The HCl and resulting solution were mixed using ultrasonication in a bath sonicator for 10 min. Finally, The fGO was seprated from solution through cerntfuagatoion at 10,000 RPM for 2 h. Fig. 1 shows the morphology of GO before and after functionalization.

#### 2.2.2 Characterization and Testing of fGO membranes

To study the fGO membranes, several tests were carried out such as DMA, contact angle analysis, SEM and DI water permutation fluxes.

The sample preparation of r SEM imaging of the PS-fGO MMMs include slicing them into small strips. For cross-section images, liquid nitrogen (LN) (N_2_) was used to fracture the membranes by first socking the membrane strip into water and then placing it in LN for few seconds until it cracks. This procedure sustains the shape of membrane's cross section to obtain good morphology images. After that, all the samples were sputter coated by gold to prevent the charging of samples, which would occur because of the accumulation of static electric fields [2]. The analysis was conducted using FEI Quanta 400 SEM (Thermo Fisher Scientific), with a chamber pressure of 1.3 mbar, high vacuum conditions and a voltage of 30 kV.

The contact angle anaylsis was carried using Kruse drop shape analyser DSA25. A sessile drop was used to place a droplet of DI water on the surface of membrane. The software then records and calculates the change in contact angle versus time for 5 s. The measurement of the contact angle starts once the water droplet reaches the membrane surface. Three repetations were carried measurements for each membrane and the average and standard deviation are reported.

The DMA tests were carried out using Q 800 DMA (TA Instruments, USA). Sample preparation include drying and cutting three rectangular samples of PS-fGO membranes. Three speciemens of each membrane were tested under uniaxial load while displacement sensor measures force, strain and amplitude. Young's modulus and Fracture strength parameters are obtained from stress vs. strain (%) graphs.

**Fig. 7:**
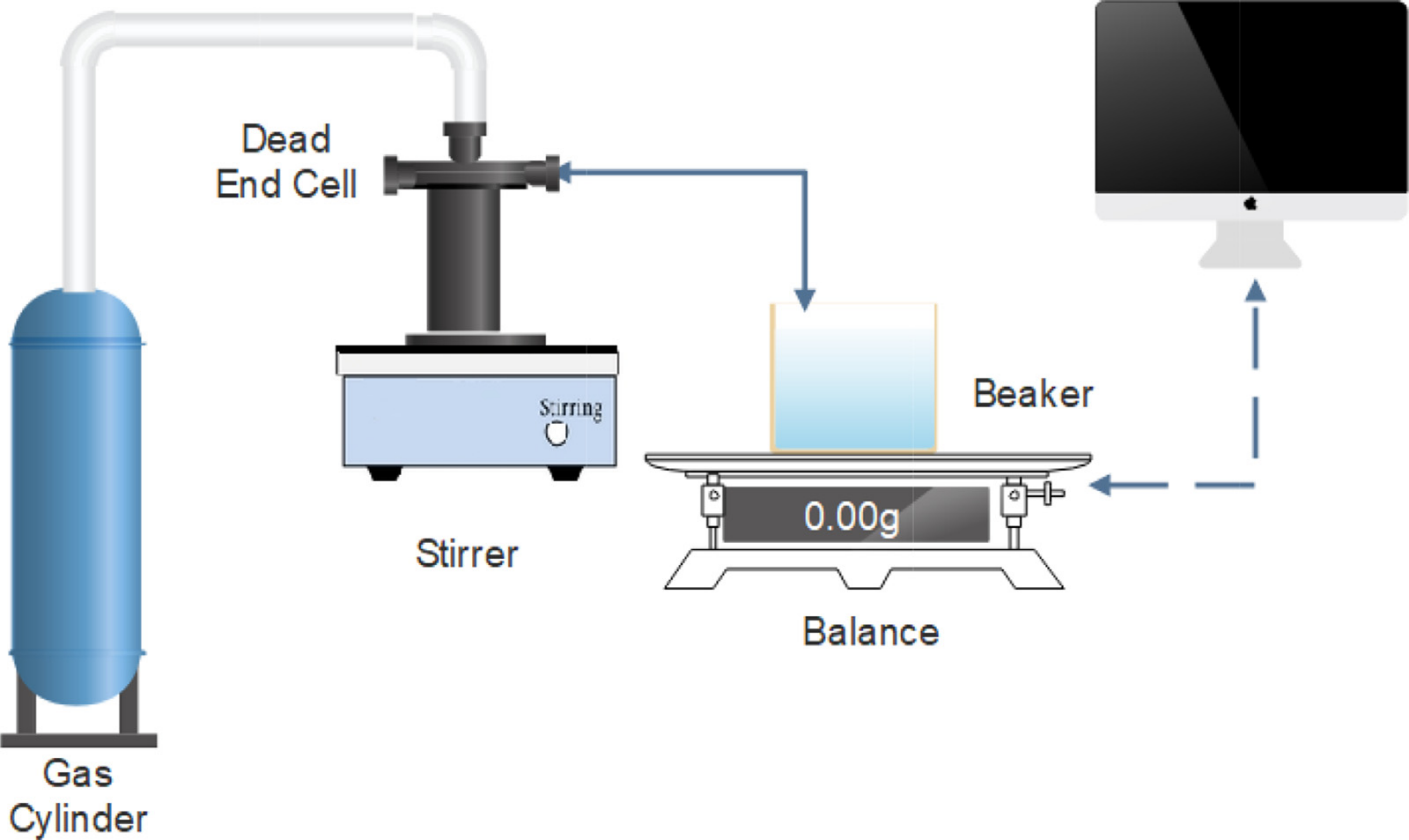
Membrane Testing Setup.

Flux measurements were carried out in a Sterlitech™ HP4750 dead-end cell under certain pressure and at room temperature. Fig. 7 shows the complete setup.; The deadend cell is connected to nitrogen (N_2_) gas cylinder that provides the necessary cross membrane of 1, 2, or 3 bar. The permeate stream flows into into a beaker placed on the top of electronic balance connected to a computer to record the mass of permeated solution versus time at time interval of 15 sThe flux (J) is calaculated as follows:

$$J=\frac{m*60}{t*A*1000}$$

where, J is the flux ((L/m^2^hr),(LMH)), m is the mass of permeated liquid (g), t is the time (min), and A is the active membrane area (14.6 cm^2^).

Fig. 2 reveals the size of the membrane pores increases to a maximum at 0.2 wt.% fGO and then starts decreasing when loading is increased. Moreover, The lowest contact angle was obtained at 0.2% of fGO loading with about 13^o^reduction. The Young's modulus and fracture strength initially increase with fGO concentration and then decrease at higher concentrations [3, 4]. Fig. 5 clearly shows that change in the mass of the collected water versus time for different memrbanes. In addition, the water flux, Fig. 6, increases to a maximum at low loadings (0.05–0.2 wt. %) and then decreases when the loading is increased further (0.4–0.8 wt. %), consistent with the previous reports [5,6]. Therefore, the addition of fGO enhanced the porosity, hydrophilicity and mechanical properties of the prepared fGO membranes as demonstrated by SEM, contact angle and DMA results. A common trend was observed that at small loadings of fGO, as discussed, characteristics of the membrane were enhanced, however, at higher loadings of fGO led to deteriorate these characteristics, due to the agglomeration of fGO. The improvement in hydrophilicity and porosity led tosignificnat improvement in the membrane permeability with about 97%%.

## Ethics Statement

This paper was not involved with any human subjects or animal experiments.

## Declaration of Competing Interest

The authors declare that they have no known competing financial interests or personal relationships that could have appeared to influence the work reported in this paper.
